# Extracellular vesicles modulate integrin signaling and subcellular energetics to promote pulmonary lymphangioleiomyomatosis metastasis

**DOI:** 10.21203/rs.3.rs-5390547/v1

**Published:** 2025-03-20

**Authors:** MAGDALENA KARBOWNICZEK, Anil Kalvala, Ashok Silwal, Bhaumik Patel, Apoorva Kasetti, Kirti Shetty, Jung-Hung Cho, Gerard Lara, Beth Daugherity, Remi Diesler, Venkatesh Pooladanda, Bo Rueda, Elizabeth Henske, Jane Yu, Maciej Markiewski

**Affiliations:** Texas Tech University Health Sciences Center; Texas Tech University Health Sciences Center; Texas Tech University Health Sciences Center; TEXAS TECH UNIVERSITY HEALTH SCIENCES CENTER; Texas Tech University Health Sciences Center; Texas Tech University Health Sciences Center; Texas Tech University Health Sciences Center; Texas Tech University Health Sciences Center; Texas Tech University Health Sciences Center; Brigham and Women’s Hospital and Harvard Medical School; Massachusetts General Hospital/Harvard Medical School; Massachusetts General Hospital; Brigham and Women’s Hospital and Harvard Medical School; University of Cincinnati College of Medicine; Texas Tech University

## Abstract

Pulmonary lymphangioleiomyomatosis (LAM) is metastatic sarcoma but mechanisms of LAM metastasis are unknown. Extracellular vesicles (EV) regulate cancer metastasis but their roles in LAM have not yet been thoroughly investigated. Here, we report the discovery of distinct LAM-EV subtypes derived from primary tumor or metastasizing LAM cells that promote LAM metastasis through ITGα6/β1-c-Src-FAK signaling, triggered by shuttling ATP synthesis to cell pseudopodia or the activation of integrin adhesion complex, respectively. This signaling leads to increased LAM cell migration, invasiveness, and stemness and regulates metastable (hybrid) phenotypes that are all pivotal for metastasis. Mouse models corroborate *in vitro* data by demonstrating a significant increase in metastatic burden upon the exposure to EV through distinct mechanisms involving either lung resident fibroblasts or metalloproteinases’ activation that are EV subtype dependent. The clinical relevance of these findings is underscored by increased EV biogenies in LAM patients and the enrichment of these EV cargo with lung tropic integrins and metalloproteinases. These findings establish EV as novel therapeutic target in LAM, warranting the future clinical studies.

## Introduction

Pulmonary lymphangioleiomyomatosis (LAM) is low-grade, understudied, and metastasizing sarcoma, predominately affecting woman, and manifesting as proliferation of tumor smooth muscle-like cells within the lungs, which ultimately leads to lung damage and failure^1–4^. It develops in association with tuberous sclerosis complex (TSC) or as a sporadic form. Both TSC and sporadic LAM result from germline or somatic *TSC1/TSC2* mutations, respectivly^5,6^ that prevent the inhibition of the mechanistic target of rapamycin (mTOR) by TSC1/TSC2 complex^7,8^. The metastatic potential of LAM, which we discovered, and the origin, possibly, from uterus, renal angiomyolipoma, or from unknown site are now well-accepted^1–4^. However, mechanisms regulating LAM metastasis remain unidentified.

EV are released from cells, including cancer cells, to mediate cell-to-cell communication, in part, through their cargo. They also promote cancer metastasis. Intercellular communication is pivotal in coordinating homeostasis, but also for pathological process. EV biogenesis and uptake are regulated by the classical and non-classical endocytic pathways^9–17^. EV released from lung cells including endothelium, pulmonary alveolar macrophages, fibroblasts, and epithelial cells contribute to asthma, chronic obstructive pulmonary disease (COPD), pulmonary hypertension, and lung cancer^18–21^. EV from epithelial cancer (carcinomas) cells and epithelial cancer stem cells (CSCs) or from the tumor microenvironment (TME) influence CSCs, premetastatic niche, metastasis, and response to therapy^22–25^. These EV transport growth factors, integrins, non-receptor tyrosine kinase protooncogene c-Src, and focal adhesion kinase (FAK), and can regulate angiogenesis, vascular permeability, premetastatic niche, and seeding of target organs by tumor cells^22–31^. EV-derived integrins (EV-ITGs) regulate anchorage-independent (i.e. in the circulation) growth of tumor cells and their organotropism^29,32^. The lung-tropic EV-ITGs: ITGα6, ITGβ4, and ITGβ1^32^, bind to the lung-resident fibroblasts and epithelial cells to promote lung metastasis via the induction of *S100*^32^. S100s promote cancer progression by altering the premetastatic niche and cancer cells^33–38^. EV also regulate cancer cell plasticity, which is linked to stemness, anoikis resistance, and increased metastatic potential^31,39–46^.

Carcinoma cells oscillate between a proliferative/differentiated and invasive/dedifferentiated phenotype (metastable/hybrid phenotypes) ^31,41–46^. Cancer cell plasticity and hybrid metastable phenotypes are also observed in non-epithelial tumors^39,40,47–52^. Sarcoma CSC regardless of origin, form clusters or sarcospheres in the circulation^3,52–55^ and share stem cell characteristics such as nestin and CD44 expression and high levels of active aldehyde dehydrogenase (ALDH)^52,53^. CD44 associates with metastable phenotypes of mesenchymal tumors^47^. LAM cells express several CSC markers including CD44, ITGs and ALDH^3,4,54–58^ and “stem-like state” LAM cells’ subpopulation exists^58^.

Despite advancements in understanding roles of EV in carcinomas, their functions in non-epithelial malignancies, especially sarcomas, including LAM, are understudied. Limited evidence defines potential roles for EV in regulating angiogenesis in non-epithelial tumors, and adhesion and migration of non-epithelial/mesenchymal malignant cells^59–63^. We previously reported that EV from *Tsc1*–null neuronal progenitors block differentiation of recipient wild-type progenitors via the activation of Notch1/mTOR pathways, phenocopying *Tsc1*-null cells, and that mTORC1 hyperactive LAM surrogate cells secrete EV, thereby affecting target cells via activation of Notch1/mTOR^64^. Consistently, LAM surrogate cells have increased EV biogenesis and cargo that enhance VEGF secretion and viability of recipient fibroblast^65^.

The release of distinct EV subtypes from the same cancer cells and mechanisms in volved in cancer progression mediated by these different EV subtypes have not been reported. We discovered that primary tumor LAM cells and metastasizing LAM cells, despite identical genotypes, release functionally different EV. Thus, our aim is to unveil heterogeneity of distinct EV populations and their significance for promoting LAM metastasis.

## Results

### EV biogenesis is increased and plasma EV cargo modified in LAM patients compared to healthy donors.

**Figure 1:**
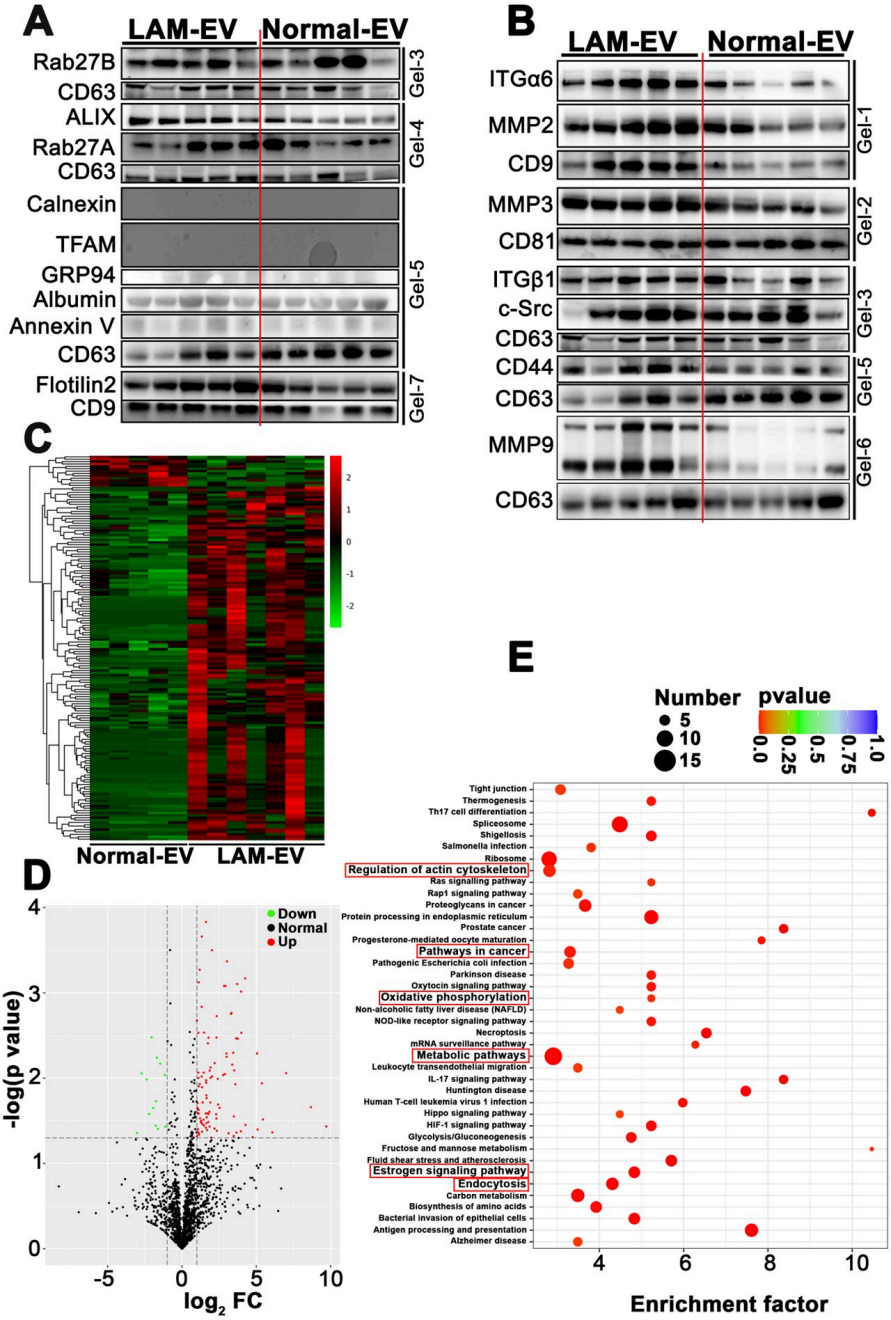
LAM-EV have modified cargo compared to Normal-EV. (A-B) Immunoblots of EV from plasma of LAM patients (LAM-EV) (n=5) and healthy donors (Normal-EV) (n=5). (A) EV associated proteins and *JEV*controls. (B) Targeted analysis of EV cargo (n=5). (C-E) Proteome of LAM-EV (n=8) and Normal-EV (n=5; for 3 patients EV were isolated from different aliquots and analyzed separately). (C) The hierarchical clustering heat map of differentially expressed proteins in EV. (D) Volcano plot and (E) top 40 enriched pathways. The same EV loading controls were used in panel A and B as data were split in between these panels.

We isolated EV from plasma of LAM patients (LAM-EV) and healthy age- and sex-matched donors (Normal-EV), using ultracentrifugation and 30% sucrose method, and analyzed by direct light scattering (DLS), fluorescent activated cell sorting (FACS), and Western immunoblotting. LAM-EV were more frequent within size range of 0–50 nm compared to Normal-EV (Suppl. Figure 1A). EV fractions from both cohorts were negative for mitochondria (TFAM), endoplasmic reticulum (ER, GRP94, Calnexin), or apoptotic bodies (Annexin V) contaminants (Fig. 1A) and expressed CD9, CD63, and CD81 EV markers (Fig. 1A and 1B, Suppl. Figure 1B). The endocytic origin of EV is supported by expression of Rab27A/B, flotillin2, and ALIX (Fig. 1A). Importantly, LAM-EV have increased expression of the majority of EV-associated proteins such as Rab27A/B, ALIX, and CD9 compared to Normal-EV (Fig. 1A and 1B), supporting increased EV biogenesis in LAM.

LAM is a low-grade metastasizing sarcoma^1^ and EV-derived integrins regulate tumor cells organotropism^29,32^ with lung-tropic EV-ITGα6/β1/β4^32^ binding to the lung-resident fibroblasts and epithelial cells to promote lung metastasis^32^. Therefore, we examined the expression of ITGα6/β1 in EV. We assessed expression of several metalloproteinases, CD44, and c-Src, as all are implemented in LAM pathogenesis^56,66–70^. We found increased expression of ITGα6/β1, MMP2, MMP3, MMP9, c-Src, and CD44 in LAM-EV compared to Normal-EV (Fig. 1B), suggesting a role of LAM-EV in lung tropism, metastasis, and disease progression. To gain insights into the functions of LAM-EV, we compared the proteome of LAM- and Normal-EV. The total of 2289 EV proteins were identified, 149 and 13 were upregulated or downregulated in LAM-EV relative to Normal-EV, respectively (Fig. 1C and 1D). Kyoto Encyclopedia of Genes and Genomes (KEGG) pathway analysis identified top 40 enriched pathways for differentially expressed proteins (DEP) in LAM-EV, including the regulation of actin cytoskeleton, pathways in cancer, oxidative phosphorylation, metabolic pathways, estrogen signaling pathway, and endocytosis (Fig. 1E).

### The loss of TSC1/2 alters EV biochemical and physical characteristics and leads to EV proteins’ enrichment similar to LAM-EV.

**Figure 2:**
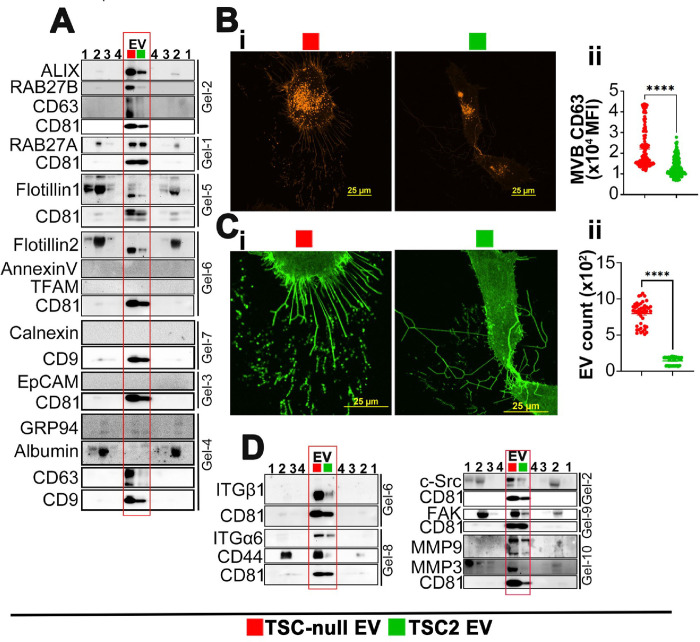
Loss of TSC1/2 increase EV biogenesis. (A) Representative immunoblots of EV associated proteins and *JEV* controls from conditioning media of 621–101 and TSC2 addback cells (n=3). (B-C) Immunofluorescence of adherent 621–101 cells expressing pHluo_M153-CD63-mScarlet (n=3). (B-i) red fluorescence indicates CD63 MVB expression, (B-ii) quantification of B-i (Two-tailed unpaired t-test, t=10.90, df=302, p<0.0001). (C-i) green fluorescence indicates CD63+EV deposited on ECM, (C-ii) quantification of C-i (Two-tailed unpaired t-test, t=26.44, df=91, p<0.0001). Data are Mean ± SEM of 3 independent experiment. (D) Representative immunoblots of targeted EV protein expression and *JEV* controls from conditioning media of 621–101 and TSC2 addback cells (n=3). ****P<0.0001. The same EV loading controls were used in panel A and D as data were split in between these panels. (1–2 SEC fractions 5 and 2, respectively; 3-conditioned media, 4- EV-depleted media).

LAM results from *TSC1/TSC2* loss of function mutations^5,6^, therefore, to corroborate patient data, we determined the impact of TSC1/2 loss on biochemical and physical EV properties and cargo. EV from TSC-null 621 – 101 LAM surrogate cells (TSC-null EV) and from isogenic control TSC2 addback cells (TSC2 EV)^64^ (Suppl. Figure 2A) were isolated and characterized. The particle concentration of TSC-null EV and TSC2 EV, isolated by ultra filtration (UF)^71^ and size exclusion chromatography (SEC) and analyzed by NTA, was 9.8×10^9^ and 7.0×10^9^ (particles/ml), respectively (Suppl. Figure 2B-i). The total particle concentration of TSC-null EV and TSC2 EV was 5.7×10^11^ and 4.52×10^11^ (particles/ml), respectively. The mean size of TSC-null EV and TSC2 EV was 105.6 ± 1.8 nm and 114.6 ± 2.0 nm, respectively. TSC2 EV were more frequent within the size range of 0–99 nm compared to TSC-null EV (Suppl. Figure 2B-ii). By DLS, the TSC-null EV were more frequent within the size range of 100–150 nm compared to TSC2 EV (Suppl. Figure 2C-i). Thus, loss of TSC2 alters EV concentrations and size distribution with TSC2-null EV being more concentrated. Zeta potential analysis indicated negative charge of EV, confirming the lack of aggregates and preservation of functionality (Suppl. Figure 2C-ii). Both types of EVs express CD63 and CD9 by FACS (Suppl. Figure 2D). The transmission electron microscopy (TEM) revealed cup-shaped morphology of EV (Suppl. Figure 2E). Western immunoblotting of EV and *JEV* controls^72^, loaded in the equal protein quantities, confirmed EV expression of tetraspanins CD9, CD63, and CD81 (Fig. 2A). EV preparations were negative for albumin, mitochondria (TFAM), endoplasmic reticulum (ER, GRP94, Calnexin), or apoptotic bodies (Annexin V) contaminants (Fig. 2A). The endocytic origin of EV is supported by expression of Rab27A/B, ALIX, and otillin-1/2 (Fig. 2A). Similar to LAM EV, TSC-null EV have increased expression of majority of EV-associated proteins, including ALIX, Rab27B, CD9, CD63, CD81, and otillin1/2 (Fig. 2A), indicating that loss of TSC2 increases EV biogenesis.

To track CD63^+^ EV, we used CD63 dual-color reporter pHluo_M153-CD63-mScarlet^73^ in 621 – 101 and TSC2 addback cells. This construct exhibits red fluorescence under acidic (e.i. in multivesicular bodies (MVB) or dual (green and red) fluorescence in neutral conditions (e.i. in secreted EV)^73^. TSC-null cells have increased intracellular/MVB expression of CD63 compared to TSC2 addback cells (Fig. 2B), suggesting increased CD63 sorting to TSC-null MVB and EV. Since TSC2 loss affects biogenesis of fluid phase EV (Fig. 2A and Suppl. Figure 2), we examined impact of TSC2 loss on EV deposited on extracellular matrix (ECM) using the same reporter. The loss of TSC2 increases EV deposition on ECM compared to TSC2 addback cells (Fig. 2C). Similar to LAM EV, TSC-null EV isolated from adherent and cultured for 72 hours 621 – 101 cells, using UF^71^ and SEC, are enriched with ITGα6/β1, CD44, c-Src, FAK, MMP9, and MMP3 (Fig. 2D).

### TSC-null EV enhance CSCs and metastable phenotypes of 621 – 101 spheres.

**Figure 3:**
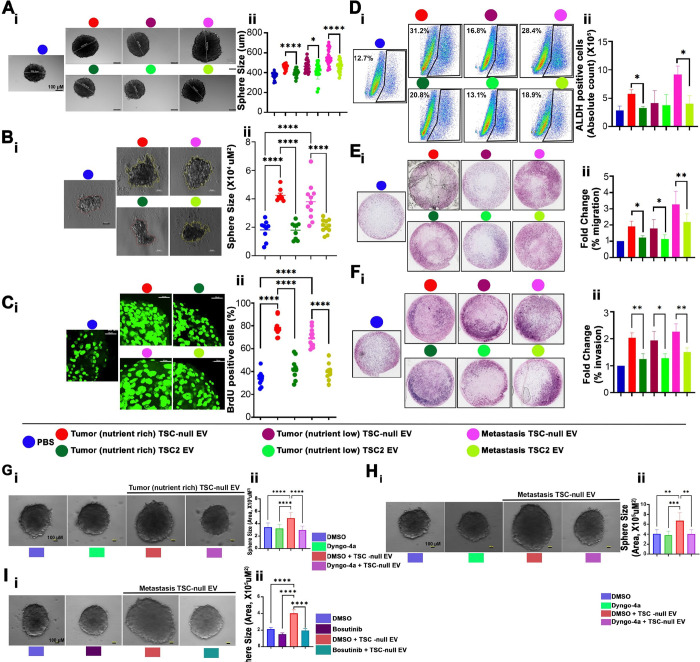
TSC-null EV enhances CSCs and metastable phenotypes of 621–101 cells. (A-B) Sphere assay (n=3); (A) Primary and (B) secondary 621–101 spheres exposed to indicated EV subtypes. (A-i, B-i) LAM patients’ specimens by (A-ii, B-ii) quantification of A-i (one-way ANOVA, F=19.60, df=6)and B-i (one-way ANOVA, F=20.8, df=4). (C)Immunofluorescence of BrdU-positive 621–101 sphere cells exposed to indicated EV subtypes (n=3) (one-way ANOVA, F=63.64, df=4). The image is from single Z stack of 1 μm each. (D) ALDEFLUOR assay; (D-i) FACS analysis of ALDH-positive 621–101 sphere cells exposed to indicated EV subtypes, (D-ii) The absolute count of ALDH-positive cells is represented in the bar graph (n=4 for PBS group, n=3 for all remaining,) One-tailed unpaired t-test, p=0.0144 (t=3.345, df=4) and p=0.0345 (t=2.469, df=4) respectively. (E-F)Transwell (E) migration (n=4) and (F) invasion (n=3) assay of 621–101 spheres exposed to indicated EV subtypes; (E-i, F-i) Representative images and (E-ii, F-ii) quantification of E-i (one-way ANOVA, F= 21.34, df=6) and F-i (one-way ANOVA, F=17.60, df=6) as a fold change in number of cells relative to PBS. (G-I) 621–101 spheres exposed or not to indicated EV subtypes in the presence of DMSO or (G-H) EV uptake inhibitor, Dyngo4a (10 μM) (n=3), or (I) c-Src inhibitor, Bosutinib (1 μM) (n=3). (G-I-i) Representative images and (G-I-ii) quantification of G-I-i. (G-ii) One-way ANOVA, F=29.20, df=3. (H-ii) One-way ANOVA, F=9.944, df=3. (I-ii) One-way ANOVA, F=46.22, df=3. *P < 0.05, **P < 0.01, ***P < 0.001, ****P < 0.0001.

TSC-null EV and TSC2 EV were isolated from adherent 621 – 101 (TSC-null) or TSC2 addback cells, respectively, grown for 3 (nutrient rich environment) or 7 (nutrient low environment) days. EV from adherent cells experimentally represent EV released from primary tumor (tumor EV). Although this 2D model does not ideally recapitulate primary tumor environment, it ensures pure fraction of tumor derived EV that are not contaminated with EV released from other cell types, thus their function can be experimentally tested. To generate EV, mimicking EV released from metastasizing/circulating LAM cells (metastasis EV), we isolated EV from 621 – 101 or TSC2 addback, floating in culture media, spheres grown for 7 days in ultra-low attachment plates, as they mimic micrometastases or circulating tumor cell spheroids^74,75^. Impact of different EV subtypes on LAM (621 – 101) cell CSC-like phenotypes was determined, using primary and secondary sphere formation, proliferation, aldehyde dehydrogenase activity (ALDH), and sphere cell migration and invasion assays. TSC-null EV subtypes increase CSCs properties of 621 – 101 spheres to a greater extent than TSC2 EV, as indicated by increased diameter of primary spheres (Fig. 3A), ability to form secondary spheres (Fig. 3B), cell proliferation (Fig. 3C), ALDH activity (Fig. 3D), increased sphere cell migration (Fig. 3E), and invasion (Fig. 3F). Interestingly, metastasis TSC-null EV led the greater increase in sphere size, ALDH activity, and sphere cell migration compared to tumor TSC-null EV subtype (Fig. 3A and 3D–E). The treatment of 621 – 101 spheres with inhibitors of EV uptake or biogenesis (Suppl. Figure 3A) reduced size (Fig. 3G, 3H, and Suppl. Figure 3B) and the migration of cells out of spheres (Suppl. Figure 3C), respectively. Similar to inhibitors of EV uptake and biogenesis, the inhibition of c-Src in these spheres (Suppl. Figure 3D) reduced sphere size (Fig. 3I) and the migration of cells of out spheres (Suppl. Figure 3E).

Consistently with sphere data, tumor TSC-null EV increase adherent 621 – 101 cells’ migration (Suppl. Figure 4A), which is associated with increased expression of ITGα6/β1, activation of c-Src, indicated by increased Y416 phosphorylation, c-Src- and integrin-mediated activation of FAK, indicated by increased phosphorylation of Y576/577 and Y397, respectively, and AKT, indicated by S473 phosphorylation in migrating cells (Suppl. Figure 4B). The activation of ITGα6/β1-c-Src-FAK-AKT axis associates with increased actin polymerization and activation of paxillin, indicated by increased F-actin expression and Y118 phosphorylation, respectively (Suppl. Figure 4C). Tumor TSC-null EV mediated activation of paxillin also results in increased co-localization of paxillin with F-actin (Suppl. Figure 4C). Finally, this TSC-null EV subtype increases invasion of 621 – 101 cells compared to TSC2 EV (Fig. 5D). The treatment of 621 – 101 cells with inhibitors of EV uptake or biogenesis (Suppl. Figure 4E-G), or c-Src (Suppl. Figure 4N) prevents TSC-null EV mediated increase in 621 – 101 cells’ migration (Suppl. Figure 4H, 4J, 4L, 4O) and invasion (Suppl. Figure 4I, 4K, 4M, 4P).

Collectively, these data suggest functional heterogeneity of TSC-null EV subtypes with sphere-derived EV having the greatest potential to enhance CSCs and metastable phenotypes of LAM cells and implicate ITGα6/β1-c-Src-FAK axis in mediating these phenotypes.

### Shuttling ATP synthesis to pseudopodia or activation of integrin adhesion complex signaling drive TSC-null EV subtypes mediated CSC metastable phenotypes of LAM cells.

**Figure 4:**
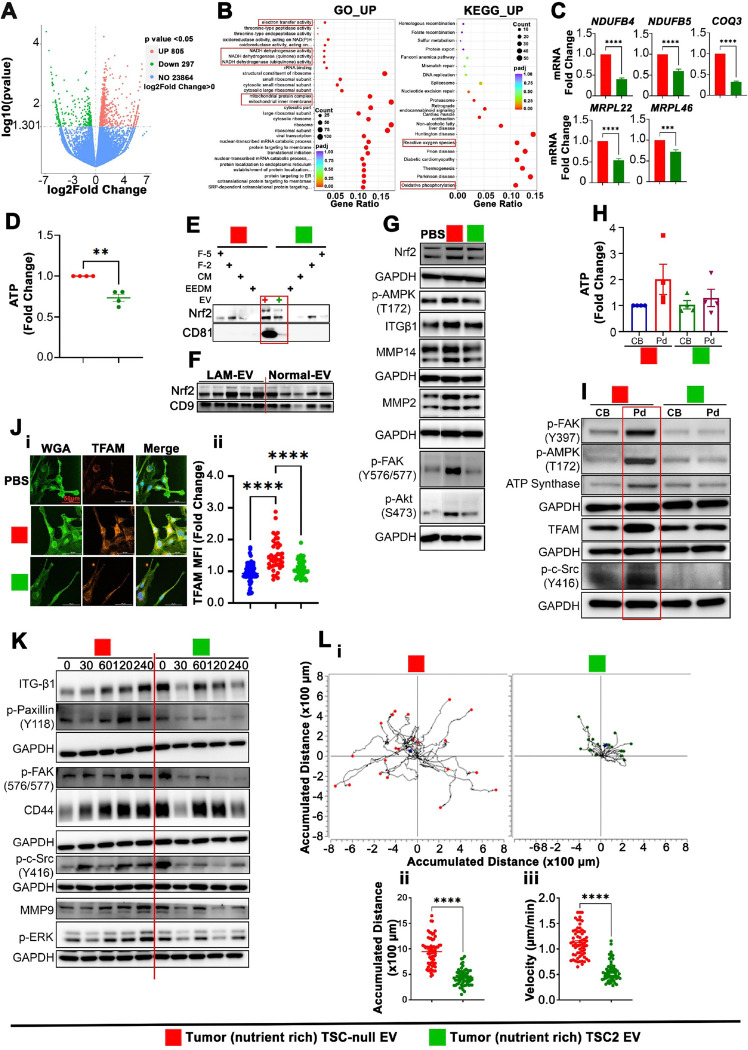
Tumor TSC-null EV promotes pseudopodia localized ATP synthesis to increase TSC-null sphere cellmigration. (A-B) RNA-Seq analysis of 621–101 spheres exposed to tumor TSC-null EV in comparison to TSC2 EV (n=3). (A) Volcano plot representing the downregulated and upregulated genes in 621–101 spheres exposed to tumor TSC-null EV in comparison to TSC2 EV. (B) Top enriched GO and KEGG pathways in the upregulated genes in 621–101 spheres exposed to tumor TSC-null EV in comparison to TSC2 EV. (C) OXPHOS and mitochondria inner membrane related genes by RT-qPCR (n=3). Two-tailed unpaired t-test, p<0.0001 (t=18.98, df=10 for *NDUFB4*), p<0.0001 (t=8.661, df=10 for *NDUFB5*), p<0.0001 (t=36.11, df=10 for *COQ3*), p<0.0001 (t=10.62, df=10 for *MRPL22*), and p=0.0003 (t=5.307, df=10 for *MRPL46*). (D) Cellular ATP level in 621–101 spheres exposed to indicated EV (n=4). Two-tailed unpaired t-test, p=0.001 (t=5.946, df=6) (E-F) Immunoblot of (E) TSC-null EV and TSC2 EV (n=3) or (F) LAM-EV (n=5) and Normal EV (n=5). The loading control from panel F was also used in Figure 1A–B as data were split in between these panels. (G) Immunoblot of 621–101 spheres exposed to PBS or tumor EV (n=3). (H) Relative levels of ATP (per microgram of protein) in CB and Pd of 621–101 sphere cells exposed to indicated EV (n=4). (I) Expression of phospho-AMPK, phospho-FAK, phospho-Src and ATP synthase in CB and Pd of 621–101 sphere cells exposed to indicated EV by immunoblot (n=3). (J) Expression of TFAM in the leading edge and protrusive structures of migrating 621–101 sphere cells exposed to tumor TSC-null or TSC2 EV by immunofluorescence (n=3). (J-i) Representative images and (J-ii) Quantification of J-i. (one-way ANOVA, F=28.46, df=2). (K) Expression of ITGβ1, CD44, MMP9, phospho-paxillin, phospho-FAK, phospho-c-Src and phospho-ERK in migrated 621–101 spheres cells exposed to tumor TSC-null or TSC2 EV (n=3). (L) Time-lapse imaging of migrated 621–101 sphere cells exposed to tumor TSC-null or TSC2 EV (n=3). (L-i) Trajectory plots, (L-ii) accumulated distance, Two-tailed unpaired t-test, p<0.0001 (t=12.18, df=121) and (L-iii) the moving velocity, Two-tailed unpaired t-test, p<0.0001 (t=11.57, df=121) (n=3, 20 cells from each independent experiment). Bars show Mean ± SEM. **P<0.001; ***P<0.0001; ****P<0.0001.

To gain insights into the mechanisms by which different TSC-null EV subtypes influence recipient LAM CSC phenotypes, sequencing (Seq) of RNA from 621 – 101 spheres exposed to tumor or metastasis EV was performed. Tumor TSC-null EV treated 621 – 101 spheres upregulate and downregulate 805 and 297 genes, respectively, relative to TSC2 EV treated spheres (Fig. 4A and 4B). Top upregulated genes identified by Gene ontology (GO) and KEGG analyses are involved in the regulation of mitochondrial inner membrane and protein complex, NADH dehydrogenase, electron transfer activity (ATP synthesis coupled electron transport), oxidative phosphorylation (OXPHOS) and reactive oxygen species (ROS) homeostasis (Fig. 4B). RT-qPCR confirmed upregulation of mitochondrial inner membrane and protein complexes, and OXPHOS related genes (Fig. 4C). This upregulation is associated with increased levels of ATP (Fig. 4D). The upregulation of OXPHOS genes and increase in ATP are likely mediated by increased expression and delivery of critical mitochondrial function regulator, Nrf2^76^ by tumor TSC-null EV, compared to tumor TSC2 EV (Fig. 4E). Clinical significance of these data is underscored by Nrf2 enrichment in LAM-EV relative to Normal EV (Fig. 4F). In addition, we found increased whole-cell expression of Nrf2, p-AMPK, ITGβ1, MMP14, MMP2, p-FAK, and p-AKT in TSC-null EV vs. TSC2 EV treated spheres (Fig. 4G). Cumulatively, these data suggest that tumor TSC-null EV mediate metabolic reprogramming of sphere cells toward OXPHOS, likely, to enhance sphere cell migration. This notion is consistent with AMPK function in mitochondria trafficking to the leading edge and protrusive structures of the cell during migration and invasion^77^. Indeed, the assessment of subcellular energetics by measuring ATP in chemotactic (FBS) pseudopodia (Pd) and cell bodies (CB), using transwell-like cell culture inserts^77^, revealed higher levels of ATP, p-AMPK, ATP synthase, TFAM, activated FAK, and c-Src in Pd compared to CB of spheres treated with tumor TSC-null EV (Fig. 4H and 4I), suggesting EV mediated shuttling of ATP synthesis to Pd. Analysis of Pd and CB of spheres treated with TSC2 EV showed reversed phenotypes (Fig. 4I). These results are corroborated by increased mitochondria presence in the leading edge and protrusive structures of migrating 621 – 101 sphere cells treated with tumor TSC-null EV vs. TSC2 EV (Fig. 4J). The TSC-null EV mediated increase in Pd ATP synthesis upregulates ITGβ1, CD44, and MMP9, and activates paxillin, FAK, c-Src, and ERK indicated by their phosphorylation in migrated sphere cells (Fig. 4K). The real-time cell tracking approach confirmed TSC-null EV-mediated increase in accumulated distance and velocity of migrated sphere cells (Fig. 4L). These data suggest that tumor TSC-null EV shift ATP synthesis to Pd to promote sphere cell migration via activation of the ITG-c-Src-FAK axis.

**Figure 5:**
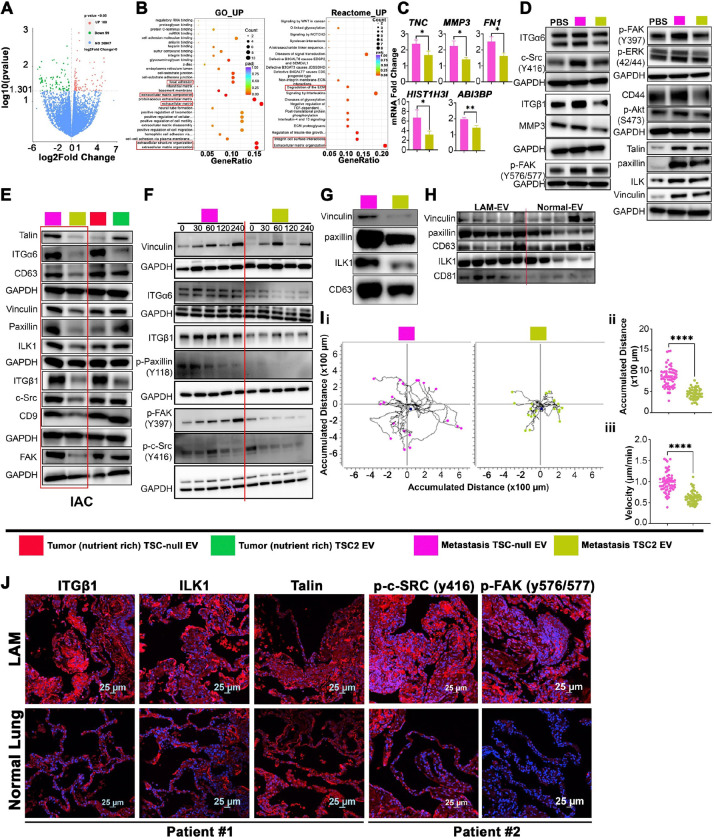
Metastasis TSC-null EV promote IAC formation to increase sphere cell migration. (A-B) RNA-Seq analysis of 621–101 spheres exposed to metastasis TSC-null EV in comparison to TSC2 EV (n=3). (A) Volcano plot representing the downregulated and upregulated genes in 621–101 spheres exposed to metastasis TSC-null EV in comparison to TSC2 EV. (B) Top enriched GO and Reactome pathways in the upregulated genes in 621–101 spheres exposed to metastasis TSC-null EV in comparison to TSC2 EV. (C) ECM receptor related genes by RT-qPCR (n=3). One-tailed unpaired t-test, p=0.035 (t=2.055, df=10 for *TNC*), p=0.0391 (t=1.961, df=10 for *HIST1H3I*) and Two-tailed unpaired t-test, p=0.0242 (t=2.652, df=10 for *MMP3*), p=0.0269 (t=2.590, df=10 for *FN1*), and p=0.0079 (t=3.311, df=10 for *ABI3BP*). (D) Immunoblot of 621–101 spheres exposed to PBS or metastasis EV (n=3). (E-F) Immunoblot of (E) IAC isolated from 621–101 spheres exposed to indicated EV, or (F) migrated spheres exposed to indicated EV (F) (n=3). (G-H) IAC protein expression in (G) metastasis TSC-null EV compared to TSC2 EV (n=3), or in (H) LAM-EV (n=5) and Normal-EV (n=5) by immunoblot. (I) Time-lapse imaging of migrated 621–101 sphere cells exposed to indicated metastasis EV (n=3). (I-i) Trajectory plots, (I-ii) accumulated distance, Two-tailed unpaired t-test, p<0.0001 (t=11.22, df=115) and (I-iii) the moving velocity, Two-tailed unpaired t-test, p<0.0001 (t=9.941, df=116) (n=3, 20 cells from each independent experiment). (J) Expression of ITGβ1, ILK1, talin, phospho-c-Src, and phospho-FAK in LAM patients’ specimens by immunofluorescence. Bars show Mean ± SEM. *P<0.01; **P<0.001; ****P<0.0001.

Metastasis TSC-null EV upregulate and downregulate 100 and 99 genes in 621 – 101 spheres, respectively, relative to spheres treated with TSC2 EV (Fig. 5A and 5B). Top upregulated genes identified by GO and Reactome analyses are involved in ECM, ECM and extracellular structure organization, ECM components, focal adhesion, integrin cell surface interaction, and ECM degradation (Fig. 5B). RT-qPCR analyses confirmed upregulation of ECM related genes (Fig. 5C). The increase in ECM gene expression in TSC2-null EV treated spheres associates with moderate increase in the whole-cell expression of ITGα6/β1, MMP3, CD44, as well as increased activation of c-Src, FAK, ERK, and AKT, indicated by their phosphorylation (Fig. 5D). TSC-null EV mediate whole-cell increase in the expression of ITGα6/β1, CD44, talin, paxillin, ILK, and vinculin, as well as c-Src and FAK activation, suggesting that these EV regulate the formation of integrin adhesion complexes (IAC)^78,79^ to promote sphere cell migration. Consistently, we found increased formation of IAC, in migrating sphere cells treated with TSC-null EV (isolated as in reference^79^), demonstrated by increased expression of ITGα6/β1 and canonical IAC proteins^78^, including talin, vinculin, paxillin, ILK, c-Src, FAK, and tetraspannins CD9 and CD63 (Fig. 5E). The TSC-null EV mediate increase in IAC formation upregulates vinculin and ITGα6/β1, and activates paxillin, FAK, and c-Src, indicated by their phosphorylation in migrated spheres (Fig. 5F). The TSC-null EV mediated upregulation of IAC signaling is likely interceded by increased expression of vinculin, paxillin, and ILK in metastasis TSC-null EV, compared to TSC2 EV (Fig. 5G). Clinical relevance of these data is corroborated by paxillin and ILK enrichment in LAM-EV relative to Normal-EV (Fig. 5H). The real-time cell tracking confirmed TSC-null EV mediated increase in accumulated distance and velocity of cells migrating from spheres (Fig. 5I). Consistently, IAC proteins, including ITGβ1, ILK1, and talin, as well as activated c-Src and FAK were expressed in LAM lesions (Fig. 5J).

These results suggest that TSC-null EV from metastasizing cells promote sphere cell migration via the activation of IAC signaling, triggered through increased IAC formation, resulting from an increased delivery of pre-formed building blocks of ILK such as vinculin and paxillin heterodimers.

### EV from TSC-null cells increase lung metastasis in a mouse model of LAM.

**Figure 6:**
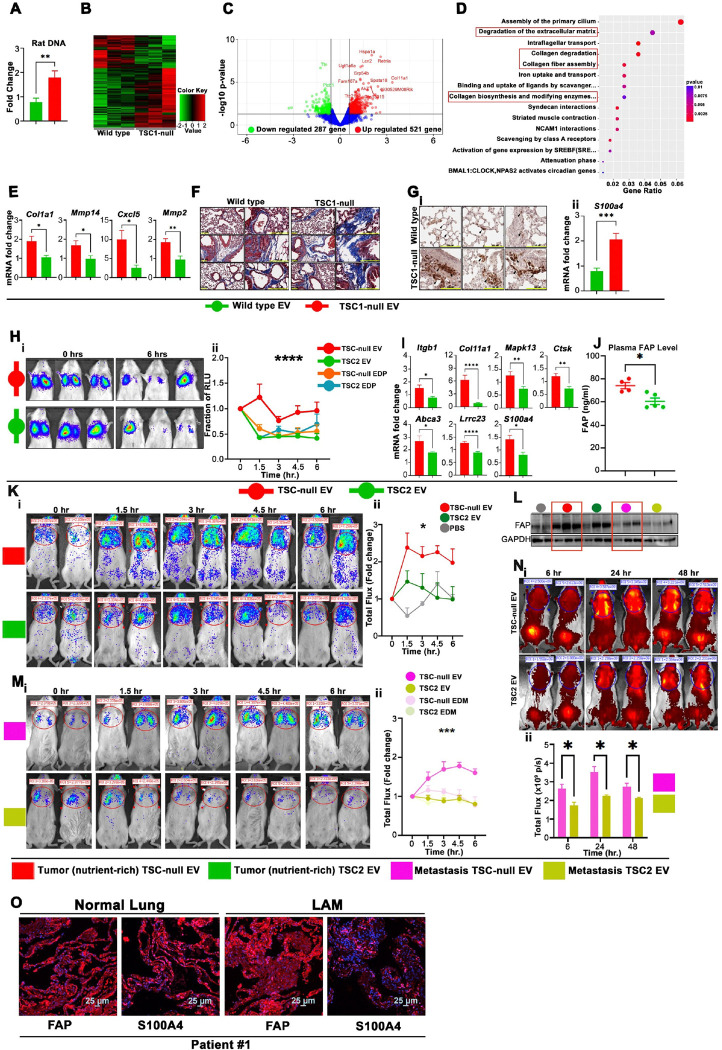
TSC-null EV promote lung seeding by LAM cells. (A) Rat DNA by qPCR (n=3).Two-tailed unpaired t-test, p=0.0058 (t=3.181, df=16) (B-D) RNA-Seq analyses (n=3). (B) Heat map, (C) Volcano plot, and (D) Top enriched Reactome pathways. (E) Expression of ECM genes by RT-qPCR (n=3). Two-tailed unpaired t-test, p=0.0135 (t=2.995, df=10 for *Col1a1*), p=0.0343 (t=2.449, df=10 for *Mmp14*), p=0.0231 (t=2.733, df=9 for *Cxcl5*), and p=0.0052 (t=3.559, df=10 for *Mmp2*) (F) Masson-Trichrome stain for collagen (blue). (G) Expression of S100A4 by (G-i) immunohistochemistry and (G-ii) RT-qPCR (n=3). Two-tailed unpaired t-test, p=0.0003 (t=4.524, df=16) (H) *In vivo* bioluminescent reporter imaging. (H-i) Bioluminescence imaging of the lungs from mice injected i.v. with 621-L9 cells 72 hr. after i.v. injection of 621L9- or TSC2-plasma EV, or controls injection and (H-ii) quantification of relative luciferase unit at different time points normalized to time zero (significance for “column factor”) (TSC-null EV n=6, TSC2 EV n=5, TSC-null EV-depleted plasma [EDP] n=4, TSC2 EDP n=3). Two-Way ANOVA, p<0.0001 for column interaction (DF=3, F=14.75). (I) ECM, epithelial, and fibroblast related genes by RT-qPCR (TSC-null EV n=6, TSC2 EV n=5 for all except for S100A4 (TSC-null EV n=6, TSC2 EV n=3). Two-tailed unpaired t-test, p=0.0124 (t=2.747, df=20 for *Itgb1*), p<0.0001 (t=5.002, df=31 for *Col11a1*), p=0.0097 (t=2.892, df=18 for *Mapk13*), p=0.0029 (t=3.416, df=19 for *Ctsk*), p=0.045 (t=1.781, df=20 for *Abca3*), p<0.0001 (t=5.192, df=18 for *Lrrc23*), and p=0.0147 (t=2.697, df=18 for *S100a4*) (J) Plasma FAP ELISA (TSC-null EV n=4, TSC2 EV n=5). Two-tailed unpaired t-test, p=0.012 (t=3.363, df=7) (K, M) Bioluminescence imaging of the lungs from mice injected i.v. with 621-L9 cells 48 hr. after (K-i) tumor TSC-null-EV or TSC2 EV (PBS n=3, TSC-null EV n=5, TSC2 EV n=4), Two-way ANOVA with Tukey’s multiple comparison test, p=0.0274 (F=7.71, DF=1) or (M-i) metastasis TSC-null-EV or TSC2 EV *vs*controls (TSC-null and TSC2 EV-depleted medium [EDM] n=3, TSC-null EV n=3, TSC2 EV n=4). Two-way ANOVA with Tukey’s multiple comparison test, p=0.0001 (F=4.701, DF=12). (K-ii, M-ii) Quantification of relative luciferase unit at different time points normalized to time zero (significance for “interaction and column factor”). (L) The lung expression of FAP from mice exposed to PBS (grey dot) or indicated EV subtypes (red boxes mark FAP expression in tumor *vs.* metastasis TSC-null EV) (PBS n=2, all other groups n=3). (N) Lung MMP activity using IVISSense MMP 750 FAST fluorescent probe from mice i.v. exposed to metastasis EV 24 hours prior to probe injection (TSC-null EV n=5, TSC2 EV n=4). (N-i) Representative fluorescent images at 6, 24, and 48 hours post-MMP probe injection, and (N-ii) quantification of fluorescence photon flux in the chest region. Two-way ANOVA with Tukey’s *multiple* comparison test, p=0.033 (F=4.396, DF=2). (O) Expression of FAP and S100A4 in LAM patient specimen by immunofluorescence. Bars and graphs show Mean ± SEM. *P < 0.05, **P < 0.01, ***P < 0.001, ****P < 0.0001.

CD9^+^CD63^+^CD81^+^ EV from *Tsc1*-null or EV from wild type E15.5 mouse embryo neuronal progenitors^64^ (Suppl. Figure 5A, 5C and 5D) were labeled and injected into the tail vein of female SCID mice 48 hr. prior to the i.v. injection of 0.5×10^6^ rat ELT3 cells (a well-characterized mouse model of LAM^80,81^). The 72 hr. after ELT3 cell injection, we found more rat DNA, reflecting metastatic burden, in the lungs of *Tsc1-*null vs. wild type EV injected mice (Fig. 6A and Suppl. Figure 5B). The RNA-Seq analyses of these lungs revealed the upregulation and downregulation of 521 and 287 genes, respectively, in *Tsc-1* null EV-vs. wild type EV-injected mice (Fig. 6B–C). *Tsc1*-null EV upregulated genes are involved in the regulation of ECM, collagen degradation, collagen biosynthesis and modifying enzymes, and collagen fiber assembly (Fig. 6D). These data were corroborated by RT-qPCR and immunohistochemistry, demonstrating increased expression of *Col1a1*, *Mmp14*, *Cxcl5*, and *Mmp2*, (Fig. 6E), collagen deposition (Fig. 6F, blue color in histology images), and increased S100A4 in the lungs of *Tsc1*-null EV compared to wild type EV treated mice (Fig. 6G). Because EV-ITGβ1/α6 activates S100A4 in lung resident cells^32^, we examined ITGβ1/α6 in *Tsc-1* null and wild type EV. *Tsc-1* null EV are enriched with ITGβ1/α6 relative to EV from wild type progenitors (Suppl. Figure 5D), consistent with the roles of these ITGs in the activation of lung fibroblasts^32^ and S100A4 in the activation of lung resident cells^32^. Next, we isolated CD9^+^CD63^+^EV from SCID/NOD mice injected i.v. with LAM patient-derived 621L9 (TSC-null) or TSC2 addback cells 6hr. prior to EV isolation (TSC-null EV *vs.* TSC2 EV) (Suppl. Figure 5E and 5F). The human tumor cell origin of EV was verified by human CD63 expression detected by anti-human antibody, which does not cross react with rodent CD63 (Suppl. Figure 5E). The treatment of tumor-free SCID/NOD mice with these plasma isolated TSC-null EV delays the clearance of 621L9 cells, injected i.v. 72 hours post EV injection, from the lungs, compared to TSC2 EV or EV-depleted plasma (Fig. 6H), and associates with increased expression of ECM, airway epithelial alveolar type 1/2, and fibroblast related genes, including *Itgβ1*, *Col11a*, *Mapk13*, *Cstk*, (ECM), *Abca3, Lrrc23* (epithelial) and *S100A4* (fibroblasts) in the lungs (Fig. 6I). The epithelial genes’ expression is consistent with gene enrichment in patient LAM-associated airway epithelial, alveolar type 1 and 2^4^. We also found increased fibroblast activating protein (FAP) in plasma of these mice (Fig. 6J).

The heterogenous functions of different TSC-null EV subtypes are supported by improved 621L9 cell lung seeding (Fig. 6K vs. 6M) and greater lung FAP expression (Fig. 6L) in tumor-free SCID/NOD mice, injected i.v. with tumor TSC-null EV (Fig. 6K), prior to tumor cells injection compared to mice injected with metastasis TSC-null EV (Fig. 6M). In contrast, metastasis EV facilitate greater activation of MMPs (Fig. 6N) compared to tumor EV (Suppl. Figure 5G), indicating different and EV subtype-dependent mechanisms facilitating LAM cell lung seeding. Although both TSC-null EV subtypes improve lung seeding by 621L9 cells compared to TSC2 EV, EDP, or PBS, (Fig. 6K and 6M), tumor EV are more efficient in supporting LAM cell retention in the lungs, suggesting greater contribution of this EV subtype to lung metastasis. Consistently, expression of FAP and S100A4 was evident in adjacent lungs in LAM patient specimen (Fig. 6O).

## Discussion

The biological significance of EV pathway in non-epithelial malignancies, especially sarcomas, including LAM, is unclear. Limited evidence supports potential roles for EV in the regulation of tumor angiogenesis, adhesion, and migration of non-epithelial/mesenchymal malignant cells^59–63^. Therefore, our study is innovative and broadens our understanding of the EV pathways in non-epithelial malignancies. Here, we provide evidence for the previously unknow functions of EV in mediating LAM progression and metastasis that are, at least partially, fueled by increased biogenesis of LAM-EV and their enrichment with proteins known to drive lung organotropic metastasis, including ITGα6/β1^32^. In addition, LAM-EV are enriched with several metalloproteinases, c-Src, and CD44 that are the establish players in LAM progression^66–70^. KEGG analyses of LAM-EV identified top enriched pathways for DEP, including regulation of actin cytoskeleton, pathways in cancer, oxidative phosphorylation, metabolic pathways, estrogen signaling pathway, and endocytosis that are known to be involved in cancer progression, thus, supporting a potential involvement of LAM-EV in LAM progression. The analysis of TSC-null EV and TSC2 EV from LAM surrogate cells and isogenic controls demonstrated that loss of TSC2 increases EV biogenesis, alters physical and biochemical properties of EV, and impacts EV cargo sorting. Thus, LAM-EV from patients share several features with TSC null EV derived from LAM surrogate cells used in the experimental settings to model LAM. Our data are consistent with previous reports indicating that ITG-β1 is enriched in EV of melanoma cells^82^ and that loss of TSC1/2 increases EV biogenesis^64,65,83,84^. Despite the impact of TSC1/2 deficiency on EV, demonstrated by our data, the long-term rapamycin treatment had no conclusive effect on EV biogenesis^65^. However, the activation of mTOR in *Tsc1/2*-null mouse embryonic fibroblasts and in hepatocytes inhibits EV release ^85^, suggesting that mTOR possibly regulates EV biogenesis in a different manner, depending on cell type, physiological vs. pathological conditions, and experimental culture methods.

The 2D and 3 D culture systems that we used in this study model, at least to some extent, - the primary tumor and metastasizing tumor cells and at the same time allow detail studies of cellular signaling. They were instrumental in the discovery of the distinct EV subtypes, reported here, and released from the genetically identical cancer cells.

**Figure 7:**
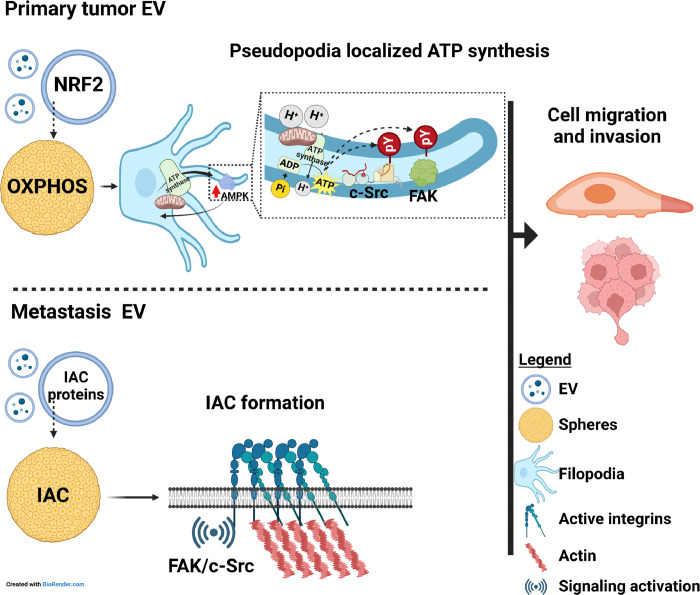
Signaling triggered by distinct EV subtypes in the recipient tumor cells.

These TSC-null EV subtypes enhance CSC and metastable phenotypes of LAM surrogate CSCs to a different magnitude and through different mechanisms. EV mimicking EV from primary tumor cells are more powerful in enhancing accumulated distance and velocity of migrated sphere cells compared to EV mimicking EV from metastasizing circulating tumor cells. Our work provide evidence for the role of primary tumor EV in regulating mitochondrial function, with respect to OXPHOS and ATP synthesis, that is increased in spheres treated with these EV prior to the initiation of migration. This EV-dependent metabolic shift is likely mediated by increased accumulation of critical mitochondrial function regulator Nrf2^76^ in EV and associates with moderate whole-cell cytoplasmic AMPK activation. Clinical relevance of these findings is underscored by Nrf2 enrichment in patient LAM-EV. This metabolic and EV mediated switch in LAM cells, associated with the enrichment of activated AMPK, TFAM, ATP synthase and increased levels of ATP in chemotactic pseudopodia, indicates novel EV function in coupling local energy demands to subcellular targeting of energy source for the activation of migratory machinery and facilitating faster and more distant cell migration. Our data are consistent with AMPK function as essential energy sensor and metabolic regulator^77^ and with AMPK mediated subcellular targeting of mitochondria to the leading edge and protrusive structures in the response to local energy demands during cell migration and invasion^77^. They are also consistent with the previous report indicating that cell protrusions of migrated cells are on high energy demand and that local AMPK activation fulfills these demands^77^. Thus, data on EV from primary tumors reveal novel and unreported function of EV in regulating plasticity of cell migration via localized AMPK activation and subcellular mitochondria and ATP synthase localization and support previous notion of heterogeneity of cellular energy balance^77^. In addition, these primary tumor EV are also more powerful in enhancing lung seeding by circulated LAM cells *in vivo* compared to EV from metastasizing cells, which is consistent with primary tumor EV superiority in promoting cell migration. While primary tumor EV promote localized ATP synthesis, and thus, faster migration, the EV from metastasizing LAM cells promote the formation of IAC through the delivery of EV pool of IAC building blocks, including vinculin, paxillin and ILK1 (Fig. 7). These IAC EV delivered building blocks have probably similar function to the cytosolic pool of these blocks^78^. The clinical relevance of this mechanism is underscored by the enrichment of paxillin and ILK1 in patient LAM-EV compared to Normal-EV. Talin, paxillin and ILK are chief determinants in this process, as increased expression of these proteins is evident in IAC, which is regulated by metastasis TSC-null EV.

Our mechanistic studies demonstrate that both TSC-null EV subtypes promote TSC-null cell migration via engaging the ITGα6/β1-c-Src-FAK-paxillin regulatory axis, which is alleviated by the blockade of EV uptake or biogenesis, or inhibition of c-Src. In integrin-dependent migration modes, different velocities come from different level of adhesion strength^86^. The slightly lower velocities and accumulated distance of migrated sphere cells treated with EV from metastasizing vs. primary tumor cells maybe be explained by the formation of IAC itself and stronger adhesions. Of note, the superiority of tumor EV over EV from metastasizing cells in promoting cell migration was alleviated when migration was examined using transwell assay and after sphere dissociation. This could be explained by dissociation procedures interfering with mitochondrial function and localization, underscoring the necessity of experimental design mimicking *in vivo* conditions. Our data are consistent with alterations in LAM surrogate cells-derived EV cargo, which enhances VEGF secretion and viability of recipient fibroblast ^65^, and with roles of EV-ITG-β1/5 and c-Src in the regulation of cell adhesion and disease progression in human osteosarcoma^87^. EV from metastasizing cells were superior in promoting stemness of LAM CSC which is consistent with role of EV of Ewing sarcoma in promoting CSC^88,89^. These data also suggest that the main role of this EV subtype is CSC protection with secondary but significant influence on CSC migration and lung seeding by circulating tumor cells. Both type of EV shared alike contribution to CSC invasion indicating their equal importance in this process.

In summary, this study reveals previously unreported heterogenous functions of EV subtypes that are derived from genetically identical primary tumor cells or metastasizing tumor cells. Engagement of these different mechanisms likely depends on differences in the tumor microenvironment (i.e. primary tumor cells/EV vs. metastasizing cells /EV). Importantly, this heterogeneity in EV functions may possibly play a role in growth of other malignancies. The reported here EV functions in the progression of LAM establish the EV pathway as new potential target for LAM therapy, warranting the future clinical studies.

## Materials and Methods

### Cell culture

We have used the following cell lines: ELT3: Tsc2-null uterine leiomyoma-derived from the Eker rat model of TSC, by C. Walker^90,91^ (from Drs. Henske and Yu); 621 – 101: human LAM surrogate cells (LAM-associated angiomyolipoma-derived) with bi-allelic *TSC2* mutations^92,93^ (from Drs. Henske and Yu); 621 – 103 (TSC2 addback): TSC2-reexpressing 621 – 101 cells (from Drs. Henske and Yu); 621L9: 621 – 101 cells stably expressing luciferase (from Dr. Yu)^94^. The cell number and viability were determined before plating. 621 – 101 and 621 – 103 were cultured in standard Dulbecco’s modified Eagle’s medium (DMEM) with the addition of 10% Fetal Bovine Serum (Corning, #35–010-CV), 1x penicillin/streptomycin (Corning, #30–002-CL), and 5 ug/ml plasmocin prophylactic (Invivogen, San Diego, CA). Plates were incubated at 37°C with 5% CO2 until cells were approximately 80% confluent. For experiments cells were plated at equal numbers in DMEM medium containing 10% FBS depleted of EV by standard ultracentrifugation^64,72^. Cells lines were routinely tested for mycoplasma. Human cell lines were STR profiled. To generate spheroids, cells were seeded on ultra-low attachment plates at density of 6000 cells/mL unless otherwise specified^95^. Briefly, cells were cultured in DMEM/F-12 (Corning, #10–090-CV) with the addition of 3% EV free FBS (Corning, #35–010-CV), 1x non-essential amino acids (Corning, #25–025-Cl), 1x penicillin/streptomycin (Corning, #30–002-CL), 1x N2 supplement (Gibco; 17502–048), 1x B27 without Vit A (Gibco, #12587–010), 20 ng/ml EGF (PROSPEC, #cyt-217), 20 ng/ml FGF (PROSPEC, #cyt-218), 10 ng/ml LIF (Peprotech, #300–05), 100 μM β-mercaptoethanol (Gibco, #21985–023) (sphere media). The E15.5 mouse embryo neural tube (NT) derived cells were cultured in 15% EV-free FBS DMEM/F12 media supplemented with 20 ng/ml EGF, 20 ng/ml bFGF, 20 ng/ml IGF, 1% B-27, 1% N2 supplement, 1% penicillin/streptomycin, as described^64,96^. We have used the following inhibitors: Bosutinib (1 μM), GW4869 (10 μM), Tipifarnib (0.1 μM) and Dyngo4a (10 μM). Inhibitors were added to day 0 and day 2 621 – 101 spheres, and spheres were allowed to grow for 7 days. Next, D7 spheres were subjected to downstream assays.

### Cell line transfection

621 – 101 and TSC2 addback cells were infected by lentiviral transduction with dual-color fluorescent reporter for CD63-positive exosome secretion and uptake (Addgene plasmid # 172118), as previously described^64^. pLenti-pHluorin_M153R-CD63-mScarlet was a gift from Alissa Weaver (Addgene plasmid #172118; http://n2t.net/addgene:172118; RRID: Addgene_172118)^73^.

### EV isolation

Ultra filtration followed by size exclusion chromatography (SEC) method: For EV isolation, adherent cells were plated in 150 mm dish at the seeding density of 5×10^6^ cells in DMEM with 10% EV free FBS and allowed to incubate for 72 hours or grown as spheres. Conditioned media was subjected to serial centrifugation steps at 500 × g (5 minutes), 2000 × g (10 minutes), and 10,000 × g (30 minutes) to remove all cell debris. Then, supernatant was filtered through 0.22 μm syringe filters and passed through pre-equilibrated Amicon 100 kDa ultra filters (Millipore Sigma, #UFC910024) using three consecutive centrifugations for 30 minutes at 3000 × g^71^. Next, concentrated EV were eluted with PBS and 100 μl of this concentrate was passed through size exclusion chromatography (SEC) columns (Cell guidance systems, #Ex03). Columns were washed several times with PBS. Six fractions were collected. The first fraction represented EV-depleted media (EDM), while the second fraction represented EV.

Ultracentrifugation combined or not with sucrose cushion method (from conditioned media or plasma): EV were purified by initial centrifugation followed by filtration (0.22 μm), standard ultracentrifugation with or without EV pelleting in density 30% sucrose gradient^72,97–99^. Equal volumes of diluted plasma (≥ 200 μL diluted in 4 mL of PBS) were used for EV isolation^72^.

#### EV characterization.

Equal quantities of initial bio-fluid, initial number of plated cells, or time of conditioning were used. The fluorescence-activated cell sorting (FACS), dynamic light scattering (DLS), and nanoparticle tracking analysis (NTA) were used^64,72^. Samples were sent to the Texas Tech University College of Arts and Sciences for transmission electron microcopy (TEM) analyses and lipid bilayer detection. Western immunoblotting assessed the presence of proteins. The EV samples and bio-fluid after EV depletion was loaded at equal quantities per *JEV*^72^. EV were examined for transmembrane and non-EV proteins (albumin), and other organelles: mitochondria, ER, and apoptotic bodies. The EV used in studies were normalized by total amount of protein in the sample. For assays, equal quantities of EV and *JEV* controls were used unless otherwise specified^72^.

EV Characterization by FACS. Isolated EV were characterized as previously described^64,100^. Briefly, aldehyde/sulfate beads (Interfacial Dynamics, Grand Island, NY, USA) were incubated with capture human CD63 (Biolegend), human CD9 (Biolegend), and mouse CD9 (BD Biosciences) or CD63 (BioLegend, San Diego, CA, USA) antibodies and then with mouse or human plasma, or conditioned media. EV-coated beads were incubated with conjugated human CD63 (Biolegend), human CD9 (Biolegend), Mouse CD63 (Biolegend), and Mouse CD9 (BD Biosciences) antibodies and analyzed by FACS.

EV Characterization by NTA. Isolated EV were analyzed by NTA (System Biosciences, version 2.3 build 2.3.5.0033.7-Beta7 of the NTA software). The EV size and particle number were evaluated.

EV Characterization by DLS using dynamic light scattering (Malvern Zetasizer ultra red, # ZSU3305), zeta size and particle distribution were evaluated. The particle distribution was reported in percent intensity defined as a plot of the relative percentage of particles in various size classes based upon the intensity of scattered light.

EV Characterization by TEM. Briefly, EVs were washed with 0.05 M Cacodylate buffer (3x) and post-fixed with 1% osmium tetroxide for 1 hour, followed by washing (3x). EVs were dehydrated through increasing ethanol concentrations (25–100%) and acetone (100%), then infiltrated with plastic (4:1, 1:1, 1:4 acetone) and embedded in Epon for 48 hours. Blocks were trimmed and mounted in a microtome to cut 1 μm thick sections, which were stained with methylene blue azure II, covered with permount, and examined under a microscope. For thin sectioning, blocks were re-trimmed, cut to 70–90 nm with a diamond knife, and placed on copper grids. Grids were stained with 1% uranyl acetate solution from a 4% stock using NERL water, washed, dried, and imaged using a Hitachi H-7650 TEM^101^.

#### EV labeling, uptake, and biogenesis.

For EV uptake, isolated EV were labeled using ExoGlow (SBI, EXOGP300A-1 or EXOGM600A-1) according to manufacturer and approximately 100 μg of EV solution was added to the cells for 6 hours. Next, cells were trypsinized and subjected for EV uptake analysis using FACS. For inhibition of EV uptake, 10 μM Dyngo4a was added 3 hours prior to EV treatment. For inhibition of EV biogenesis cells were treated with 0.1 μM Tipifarnib or 10 μM GW4869, and media was subjected for EV isolation followed by EV characterization by FACS^9,14–17,102^.

### Cell lysis

Cells were washed with ice-cold PBS and lysed on ice for 15–20 minutes with RIPA buffer supplemented with PhosSTOP^™^ (Roche, 4906845001) and protease inhibitors (Thermo scientific, A32965) for whole cell lysates (WCL). WCL were cleared by the centrifugation at 14,000 RPM for 15 min at 4°C and protein concentration was determined using the Bradford assay (Bio-Rad Laboratories, 5000006).

### Western immunoblotting

Protein lysates were boiled for 10 min and subjected to SDS-PAGE electrophoresis using 4%–12% precast gels (Invitrogen, NP0336BOX, and NP0322BOX). Primary antibody binding was detected using HRP-conjugated anti-mouse or anti-rabbit antibody (Invitrogen) and chemiluminescence (Thermo Scientific). Primary antibodies were used at a dilution of 1:1,000 in 5% BSA/TBST solution, and secondary antibodies at 1:10,000 in 5% milk/TBST unless otherwise specified (Supplementary Table 1).

### Quantitative (q) real time (RT)-PCR

RNA was extracted using Rneasy plus mini kit (Qiagen) and cDNA was generated using High-Capacity RNA-to-cDNA^™^ kit (Applied Biosystems). The qRT-PCR was performed using High Capacity cDNA Synthesis Kit, Fast SybrGreen and StepOne Plus (Applied Biosystems). The comparative Ct method (2−ΔΔCt) and RT2 profiler PCR Array Data Analysis (SAB Biosciences) was used to determine fold differences between the target gene and the housekeeping gene GAPDH. Primer sequences were established based on https://pga.mgh.harvard.edu/primerbank/ (Supplementary Table 2).

### Immunofluorescence and confocal microscopy

For adherent cells, 621 – 101 cells cultured overnight on coverslips and fixed with 4% formaldehyde in PBS for 15 minutes at room temperature, rinsed with PBS, and then exposed to blocking buffer (5% BSA/0.3% Triton-X-100 in PBS) for 1 hour at room temperature. This was followed by 1-hour incubation with F-Actin (Spirochrome, #SPY555-actin, Supplementary Table 1) followed by overnight incubation with anti-Rabbit p-paxillin (Y118) (Cell Signaling Technology, #2541S, Supplementary Table 1). The next day, the cells were rinsed with PBS and incubated with anti-Rabbit-FITC (1:400 dilution) for 1 hour at room temperature and rinsed with PBS. Cells were mounted with ProLong^™^ Diamond Antifade Mountant (Thermofischer scientific, #P36965) and imaged using Nikon AX R confocal microscope.

For sphere cells, 621 – 101 spheres were plated onto collagen-coated coverslips and allowed to migrate for 6 hours before staining with TFAM and p-paxillin (Y118) antibodies, and WGA (Supplementary Table 1). Fluorescence was observed with Nikon AX R confocal microscope and quantified using Nikon Elements Advanced Research Image-Analysis software. Data is expressed as mean fluorescence intensity (MFI) or Pearson correlation coefficient for colocalization.

### Immunohistochemistry

Sections were deparaffinized, incubated with primary antibody, S100A4 (1:800, Rabbit mAb, #13018, Cell Signaling Technology) and biotinylated secondary antibodies-Rabbit specific HRP/DAB (ABC) Detection IHC kit (#PK-4000, Vector Laboratories, Inc, CA, USA).

### Trichome Mason staining

Masson’s Trichrome staining was conducted as previously^103^.

### Scratch migration assay

621 – 101 cells were seeded at a density of 1.2 million cells per well in EV-free DMEM/F12 complete media on a 6-well plate. The following day, a scratch was created vertically down the center of each well using a comb and images were captured every 2 hours using a Citation 5. The wound healing efficiency was determined from three selected fields at each time point by calculating the difference between the original wound area and the post-migration area, divided by the original wound area.

### Transwell migration and invasion assays

Transwell chambers were coated with a 100 μl solution containing 50 μg/cm^2^ rat tail collagen IV (for migration) or 300 μg/ml growth factor reduced Matrigel matrix (for invasion), followed by a 2-hour incubation at room temperature with gentle shaking, or at 37°C in a CO2 incubator, respectively. The 0.5 ml volume of single-cell suspensions (25000–50000 cells/well) in serum-free medium were plated into 24-well inserts. The 0.75 ml of 10% FBS complete EV free DMEM/F12 media was added to companion plate wells. Chambers were incubated for 16 hours (migration) or 72 hours (invasion) at 37°C with 5% CO2. Non-migrated/invaded cells were removed from the upper chamber using cotton swabs, and remaining cells at the bottom were stained, air-dried, scanned using Aperio, and quantified using ImageJ software (Imagej 1.53k, NIH, USA). We have used the following inhibitors: Bosutinib (1 μM), GW4869 (10 μM), Tipifarnib (0.1 μM), which were added to 621 – 101 adherent cells and maintained for 24 hours. Next, cells were trypsinized and plated to transwell inserts in the presence of inhibitors and EV. Dyngo4a (10 μM) was added to 621 – 101 cells 3 hours prior to EV exposure. Cells were then incubated for 16 and 72 hours for migration and invasion, respectively.

### Primary and secondary sphere assays

Cells were seeded at density of 500 cells per well into ultra-low attachment round or flat bottom 96-well plates in sphere media. Plates were centrifuged daily at 200 × g for 5 minutes. Cells were treated with EV or inhibitors on day 0 and day 2 and grown for 7 days or as indicated. The sphere size was determined using either confocal microscopy or Citation 5. Secondary sphere formation assay was performed with slight modifications of previous protocol^104^. Briefly, on day 7, primary spheres were dissociated with trypsin, neutralized with serum media, and filtered through a 70 μm nylon mesh to form a single-cell suspension. Cell count and viability were assessed with a cell counter. Next, 500 cells per well were seeded into low-attachment 96-well plates containing sphere media. After seven days, spheres were imaged and sphere size was assessed using Nikon AX-R confocal microscope and NIS-Elements AR 5.42.03 64-bit software respectively.

### Sphere cell proliferation

Single primary spheres were labeled with EdU labeling solution at day 6 and final concentration of 10 μM of Click-iT^™^ EdU Alexa Fluor^™^ 488 (Invitrogen, #C10337) in sphere media, incubated for 4 hours, then transferred to 1.5 ml tubes, washed once with 3% BSA in PBS and fixed with 3.7% formaldehyde in PBS for 30 minutes. Subsequently, spheres were washed twice with 3% BSA in PBS and permeabilized with 0.5% Triton X-100 in PBS for 30 minutes at room temperature followed by three washes with 3% BSA in PBS. The spheres were incubated in Click-iT reaction cocktail in the dark for 30 minutes at room temperature, washed twice with 3% BSA in PBS, counterstained with Fluoro-Gel II containing DAPI (Electron Microscopy Sciences, #17985–51) for 45 minutes, washed once with 3% BSA in PBS before mounting in ProLong^™^ Diamond Antifade Mountant (Invitrogen, Catalog no P36961). Images were captured using Nikon AX-R confocal Microscope at 20x magnification, with a zoom size of 4 and 1-μm-thick Z-stacks spanning the entire sphere. The images were analyzed on ImageJ using the ‘Multi-point’ tool to count the percentage of EdU positive cells against the nuclear counterstain in the same sphere region.

### ALDH assay

The ALDH activity was measured using ALDEFLUOR^™^ Kit (StemCell Technologies, Canada).

### Sphere migration assay

621 – 101 cells were cultured in sphere-forming media and treated with EVs or inhibitors on days 0 and 2. On day 7, spheres were transferred to collagen-coated 96-well plates (50 μg/cm^2^) and allowed to settle for 2 hours, next imaged using Citation 5, then cells were allowed to migrate in a CO_2_ incubator for 24 hours imaged again. Migration was assessed by drawing 10 lines from the sphere edge to the furthest point of migrated cells using Citation 5 to quantify migrated distance. Data were analyzed with GraphPad Prism.

### Time-lapse imaging of sphere cell migration and trajectory plots generation

Day 7 spheres were transferred to a flat-bottom 96-well plate coated with 50 ug/cm^2^ of rat tail Type-I collagen (Advanced BioMatrix, #5153–100MG) allowed to settle at the bottoms for 2 hours in a CO2 incubator before time-lapse imaging using Nikon Microscope AX-R and 4x objective. Spheres were imaged every 10 minutes over 12–16 hours. The captured image had 30 pixels corresponding to 100 μm. Next, migrated cells were tracked using the ImageJ Manual Tracking plugin to determine their positional values (x, y) at each time point. The output from the Manual Tracking plugin was further processed using the Ibidi Chemotaxis and Migration Tool V2.0 to generate trajectory plots of migrating cells and determine their distance and velocity.

### Measurement of ATP level

Cellular ATP levels were assessed using the Enzylight ATP Assay Kit (BioAssay Systems, USA, #EATP-100) following the manufacturer’s protocols. 621 – 101 cells, cultured as spheres for seven days, were lysed with 50 μl of PBS. ATP was determined by the amount of light emitted after the reaction of D-luciferin and ATP catalyzed by luciferase. The luminescent signal was recorded using the luminometry mode of a plate reader (BioTek Cytation5). ATP levels were assayed and normalized to the protein content and reported as μM per mg of protein.

### Cell body and pseudopodia isolation

Pseudopods were obtained from cell bodies as described previously^77^. In brief, cell culture inserts with 3.0 μm-pore polycarbonate membranes (CELLTREAT, #230609) were coated with collagen at a concentration of 50 μg/cm^2^ for 2 hours at room temperature, rinsed with PBS, and seeded with cells from dissociated spheres. Cells were allowed to migrate for 6 hours. The pseudopods were collected by gently scraping the top surface of the insert with a cotton swab and transferred to lysis buffer or PBS. To isolate the cell bodies, the undersides of the inserts were scraped to remove the pseudopods, and the remaining cell bodies were collected into lysis buffer or PBS for subsequent analysis.

### Integrin adhesion complexes isolation

The isolation of integrin-associated adhesion complexes was carried as described previously^105^ with minor modifications. Spheres were transferred to a collagen-coated plates for 6 hours. Next, plates were washed twice with pre-warmed DMEM-HEPES to remove non-adherent cells, followed by an 8-minute incubation with a 6 mM solution of DTBP cross-linker (Thermo Fisher Scientific) in DMEM-HEPES at 37°C. The cross-linker was quenched with 150 μl of 1M Tris-HCl, pH 8. The plates were then incubated with a modified RIPA buffer for 3 minutes and washed twice with PBS. Adhesion complexes were isolated using an adhesion recovery solution. To precipitate the adhesion complex proteins, four volumes of acetone were added, and the mixture was stored overnight at −80°C. The precipitated proteins were collected by centrifugation at 16,000 × g for 20 minutes at 4°C. The pellet was washed with acetone, dried in a fume hood at room temperature for about 20 minutes, resuspended in SDS-PAGE sample buffer, and boiled before being subjected to western blotting.

### Proteomic analysis

LAM-EV and Normal-EV proteome analysis was carried out by Creative Proteomics and the total of 2289 proteins were identified. The fold-change cutoff was set when proteins with quantitative ratios above 2 or below 1/2 are deemed significant. Proteins of relative quantitation were divided into two categories. Quantitative ratio over 2 was considered up-regulation while quantitative ratio less than 1/2 was considered down-regulation. Intensive bioinformatics analyses were carried out to analyze those quantifiable proteins, including GO annotation, KEGG annotation, cluster analysis, volcano plot, and protein-protein interactions analysis.

### RNA sequencing analysis

RNA sequencing analyses were carried out by Quick Biology and Novogene. Quick Biology analyzed SCID mice-based studies. For this, the reads were first mapped to the latest UCSC transcript set using Bowtie2 version 2.1.0^106^ and the gene expression level was estimated using RSEM v1.2.15^107^. TMM (trimmed mean of M-values) was used to normalize the gene expression. Differentially expressed genes were identified using the edgeR program^108^. Genes showing altered expression with p < 0.05 and more than 1.5-fold changes were considered differentially expressed. Goseq^109^ was used to perform the GO enrichment analysis and Kobas^110^ was used to performed the pathway analysis.Novogene analyzed 621 – 101 sphere-based studies. For this, sample quantification, integrity and purity were checked by using Agilent 5400 instrument. Messenger RNA was purified from total RNA using poly-T oligo-attached magnetic beads. After fragmentation, the first strand cDNA was synthesized using random hexamer primers, followed by the second strand cDNA synthesis using either dUTP for directional library or dTTP for non-directional library^111^. The library was checked with Qubit and real-time PCR for quantification and bioanalyzer for size distribution detection. Quantified libraries were pooled and sequenced on Illumina platforms, according to effective library concentration and data amount. Raw data (raw reads) of fastq format were firstly processed through in-house perl scripts. In this step, clean data (clean reads) were obtained by removing reads containing adapter, reads containing ploy-N and low-quality reads from raw data. At the same time, Q20, Q30 and GC content the clean data were calculated. All the downstream analyses were based on the clean data with high quality. Reference genome and gene model annotation files were downloaded from genome website directly. Index of the reference genome was built using Hisat2 v2.0.5 and paired-end clean 1 reads were aligned to the reference genome using Hisat2 v2.0.5. The Hisat2 was selected as the mapping tool for that Hisat2 can generate a database of splice junctions based on the gene model annotation le and thus a better mapping result than other non-splice mapping tools^112^. The featureCounts v1.5.0-p3 was used to count the reads numbers mapped to each gene^113^. Then, FPKM of each gene was calculated based on the length of the gene and reads count mapped to this gene. FPKM, expected number of Fragments Per Kilobase of transcript sequence per Millions base pairs sequenced, considers the effect of sequencing depth and gene length for the reads count at the same time, and is currently the most commonly used method for estimating gene. (For DESeq2^114^ with biological replicates) Differential expression analysis^115^ of two conditions/groups (two biological replicates per condition) was performed using the DESeq2Rpackage (1.20.0). DESeq2 provide statistical routines for determining differential expression in digital gene expression data using a model based on the negative binomial distribution. The resulting P-values were adjusted using the Benjamini and Hochberg’s approach for controlling the false discovery rate. Genes with an adjusted P-value < = 0.05found by DESeq2 were assigned as differentially expressed. (For edgeR^108^ without biological replicates) Prior to differential gene expression analysis, for each sequenced library, the read counts were adjusted by edgeR program package through one scaling normalized factor. Differential expression analysis of two conditions was performed using the edgeR R package (3.22.5). The P values were adjusted using the Benjamini & Hochberg method. Corrected P-value of 0.05 and absolute foldchange of 2were set as the threshold for significantly differential expression. Gene Ontology^109^ (GO) enrichment analysis of differentially expressed genes was implemented by the cluster Profiler R package, in which gene length bias was corrected. GO terms with corrected P-value less than 0.05 were considered significantly enriched by differential expressed genes. KEGG is a database resource for understanding high-level functions and utilities of the biological system, such as the cell, the organism and the ecosystem, from molecular-level information, especially large-scale molecular datasets generated by genome sequencing and other high-through put experimental technologies (http://www.genome.jp/kegg/). The clusterProfiler R package was used to test the statistical enrichment of differential expression genes in KEGG^116^ pathways. The Reactome database brings together the various reactions and biological pathways of human model species. Reactome pathways with corrected P-value less than 0.05 were considered significantly enriched by differential expressed genes. The DO (Disease Ontology) database describes the function of human genes and diseases. DO pathways with corrected P-value less than 0.05were considered significantly enriched by differential expressed genes. The DisGeNET database integrates human disease-related genes. DisGeNET pathways with corrected P-value less than 0.05 were considered significantly enriched by differential expressed genes. The clusterProfiler software was used to test the statistical enrichment of differentially expressed genes in the Reactome pathway, the DO pathway, and the DisGeNET pathway. Gene Set Enrichment Analysis (GSEA) is a computational approach to determine if a pre-defined Gene Set can show a significant consistent difference between two biological states. The genes were ranked according to the degree of differential expression in the two samples, and then the predefined Gene Set were tested to see if they were enriched at the top or bottom of the list. Gene set enrichment analysis can include subtle expression changes. The local version of the GSEA analysis tool was used (http://www.broadinstitute.org/gsea/index.jsp), GO, KEGG, Reactome, DO and DisGeNET data sets were used for GSEA independently.

### Rat DNA quantification

Rat DNA in SCID mice lungs was quantified as previously described^117^.

### FAP ELISA

Mouse plasma FAP levels were determined by solid phase sandwich ELISA according to the manufacturer’s instructions using DuoSet Mouse FAP (R & D Systems, USA #DY8647–05).

### Animal studies

For ELT3-based studies, female CB17/Icr-*Prkdc*^*scid*^/IcrlcoCrl (CB17 SCID/Fox Chase SCID) mice at 4–6 weeks of age were purchased from Charles River Laboratories. For 621–101-based studies, female NOD-Prkdc^em26Cd52^Il2rg^em26Cd22^/NjuCrl (NCG) mice at 7–8 weeks of age were purchased from Charles River Laboratories.

For short-term lung colonization using SCID mice and neural tube derived EV. The neural tube (NT) from an E15.5 embryo was collected from both *Nestin-Cre*^*+*^*Tsc1*^*−/−*^ and wild-type littermates and cultured as described^64,96^. EV were purified from the culture media using UC method^9^, labeled with AF488 ExoGlow, and tail vein injected to SCID mice. 48 hours post EV inoculation 5 × 10^5^ of ELT3 cells were tail vein injected and mice were harvested 72 hours later.

For short-term lung colonization using NCG mice and plasma derived EV. 5 × 10^5^ of 621-L9 and TSC2 addback cells in 100 μL of PBS were tail vein injected into NCG mice. Six hours later, mice were harvested and plasma EV were isolated using 30% sucrose cushion and UC method, and labeled with AF488 ExoGlow. Approximately equal amount of EV or EDP protein were tail vein injected to tumor-free NCG mice in the first experiment, whereas equal amount of EV protein and higher amount of EDP protein were tail vein injected in the second experiment. 72 hours post EV or EDP inoculation, mice were tail vein injected with 5 × 10^5^ of 621-L9 luciferase cells and imaged using IVIS bioluminescence imaging system as previously described^80^.

For short-term lung colonization using NCG mice and conditioning media derived EV. NCG mice were tail vein injected with 3 μg of EV, EDM, or PBS. 48 hours post EV, EDM, or PBS inoculation mice were tail vein injected with 5 × 10^5^ 621-L9 luciferase cells and imaged using IVIS bioluminescence imaging system as previously described^80^.

### In vivo bioluminescent reporter imaging

Ten minutes before imaging, mice were given D-luciferin (120 mg/kg, i.p., PerkinElmer Inc, 122799). Bioluminescent signals were recorded using the IVIS Spectrum System. Total photon flux of chest regions was analyzed and quantified.

### In vivo MMP study

The IVISense MMP-750 FAST Fluorescent Probe (MMPSense) from Revvity was used following the manufacturer’s instructions. The probe was reconstituted in 1.2 ml PBS and tail vein injected into NCG mice 24 hours post EV inoculation at the dose of 2 nmol in 100 μl per mouse. The fluorescent signal was recorded using IVIS imaging system.

#### Statistics.

Data are expressed as mean ± SEM. Grubbs’ test was used to identify outliers. The significance of differences between groups were assessed using either a two-tailed Student’s t-test, one-way ANOVA, or two-way ANOVA, as appropriate. Post-hoc comparisons were conducted with Tukey’s multiple comparison test. Differences were considered statistically significant at p-values < 0.05. Statistical analyses were performed using GraphPad Prism, version 10.2.3.

### Study approval

Human plasma samples from LAM patients and healthy donors were from the Center for LAM Research at Brigham and Women’s Hospital with obtained informed consent from all human participants under Institutional Review Board approval.

Mouse studies were performed in compliance with the U.S. Department of Health and Human Services Guide for the Care and Use of Laboratory Animals and approved by the TTUHSC Institutional Animal Care and Use Committee (10034/22006).
